# The Beat

**DOI:** 10.1289/ehp.121-a17

**Published:** 2013-01-01

**Authors:** Erin E. Dooley

**Affiliations:** **Erin E. Dooley** is a staff writer for *EHP*.

## New EPA Criteria for Recreational Waters

The U.S. EPA has released new recommendations to help states and communities protect the health of people enjoying recreational waters including beaches and rivers.^^1^^ The new guidelines suggest pathogen thresholds to protect people from a broader range of symptoms than before. They also narrow the window for averaging sampling, which will provide a more accurate reading of water quality during the monitoring period and allow for improved communication to the public about water quality. The EPA is also making available a new rapid testing tool that can determine the safety of water quality within hours of sampling, an early-alert process for states to issue swimming advisories, and tools for predicting water quality problems, identifying pollution sources, and developing criteria for specific beaches.

## Traffic Pollution Explored as a Possible Factor in Autism

Autism spectrum disorders are estimated to affect 1 in 88 U.S. children.^^2^^ Although genetic factors are thought to play a part in the development of such disorders, environmental links also are suspected. In one recent case–control study of potential links between autism and air pollution, researchers found that autistic children were more likely than control children to have been exposed to the highest quartile of air pollution during gestation and the first year of life.^^3^^ The observations don’t prove a causal link between air pollution and autism, but do point toward potential pathways to be explored in later studies.

## Mexican State Launches AIRNow–International

With the support of the Commission for Environmental Cooperation, Nuevo León is now the first Mexican state to use the AIRNow–International air quality information management system—an early step in integrating the systems used across North America to process and disseminate information on ambient air quality.^^4^^ Once the system is fully operational, the state will be able to exchange data more quickly and reliably with Mexico’s national air quality monitoring program, process data in an automated fashion, and provide the public and decision makers with near real-time information on air quality conditions. The system also provides the state with tools to evaluate pollution control strategies, track pollutant movement, and forecast air pollution events.

## A Dog’s Nose Knows

Diarrhea caused by *Clostridium difficile* infection has a distinctive odor. Now Dutch researchers have demonstrated it may be possible to train dogs to sniff out *C. difficile* infections in stool samples and hospital environments.^^5^^ After two months of training, a beagle named Cliff was able to correctly identify 100% of *C. difficile*–positive samples and 94% of negative samples. *C. difficile* infections most commonly affect older hospital patients and elder-care residents who have been taking antibiotics. Infection can sometimes progress to a life-threatening inflammation of the bowel, and early detection is crucial to thwart transmission. However, conventional diagnostic testing is expensive, and treatment can be delayed by up to a week.

**Figure:**
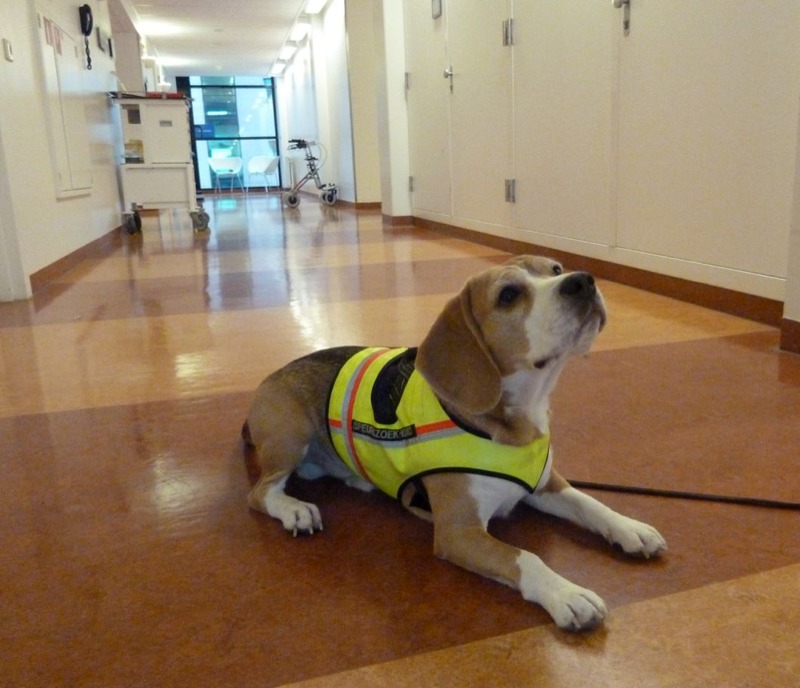
In a proof-of-concept experiment, Cliff the beagle was successfully trained to sniff out *C. difficile* infections. Bomers et al.; doi: http://dx.doi.org/10.1136/bmj.e7396

## Dispersant and Oil Mixture Is Toxic to Plankton

During the cleanup of the 2010 BP *Deepwater Horizon* oil spill, nearly 2 million gallons of dispersants were used to help keep the oil from fouling the shoreline of the Gulf of Mexico. Dispersants also make oil droplets small enough that they are bioavailable to microscopic organisms at the bottom of the food chain.

Investigators now report that crude oil mixed with Corexit 9500A^®^ dispersant at concentrations recommended for use in spills was 27–52 times more toxic to plankton than either crude oil or dispersant alone.^^6^^ However, tests of the concentration of Corexit 9500A actually used at the *Deepwater Horizon* spill produced no excess toxicity. This study is one of the first to look at the synergistic toxicity of oil and dispersant.

**Figure:**
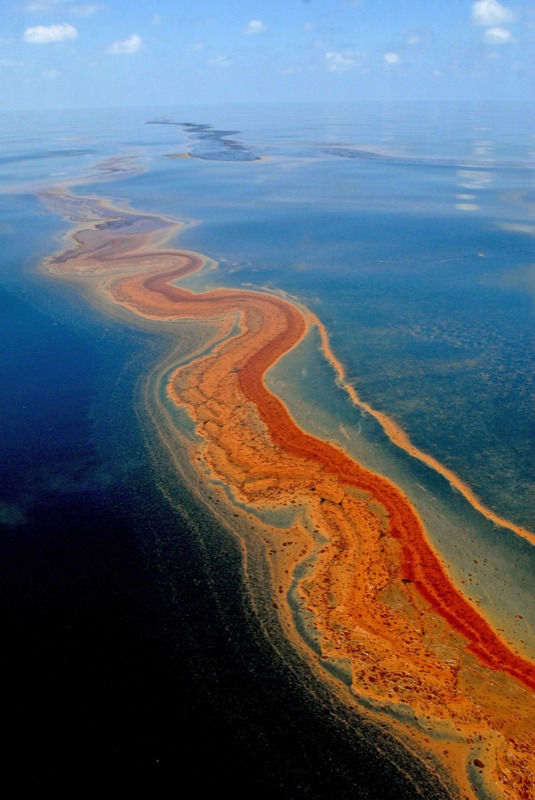
Dispersants are applied to keep clumps of oil like this from fouling shorelines after oil spills. © EPA/Christopher Berkey/Corbis
